# Heart Matters: a study protocol for a community based randomized trial aimed at reducing cardiovascular risk in a rural, African American community

**DOI:** 10.1186/s12889-018-5802-1

**Published:** 2018-07-31

**Authors:** Giselle Corbie-Smith, Crystal Wiley-Cene, Kiana Bess, Tiffany Young, Gaurav Dave, Katrina Ellis, Stephanie M. Hoover, Feng-Chang Lin, Mysha Wynn, Shirley McFarlin, Jamie Ede

**Affiliations:** 1 University of North Carolina School of Medicine, Chapel Hill, USA; 2 Project Momentum, Inc., Rocky Mount, USA; 3 James McFarlin Community Development, Inc., Rocky Mount, USA

**Keywords:** Cardiovascular disease risk, Study protocol, African Americans, Community-based participatory research, Randomized stepped wedge design

## Abstract

**Background:**

African Americans living in the rural south have the highest prevalence of cardiovascular disease (CVD) risk in the United States. Given this geographic and racial disparity, intervention implementation needs to be evaluated for effectiveness and feasibility with African Americans in the rural south.

**Methods:**

The trial developed out of a community-based participatory research partnership, Project GRACE, and community partners who are collaborators throughout the study. Heart Matters is a randomized stepped wedge trial that will assess the effectiveness of a 12-month behavioral change intervention adapted from PREMIER, an evidence-based treatment targeting multiple CVD risk factors. 140 participants will be recruited through 8 community- or faith-based organizations to participate in the intervention. Through matched pair randomization, organizations will be randomized to begin immediately after baseline data collection (Arm 1) or delayed 6 months (Arm 2). Data collection will occur at baseline, 6, 12, and 18 months. The primary outcome is change in body weight. In addition to assessing effectiveness, the study will also evaluate process and feasibility outcomes through quantitative and qualitative data collection.

**Discussion:**

This study will contribute to CVD prevention research and likely have a positive impact on the rural, African American community where the trial occurs. Our study is unique in its use of community partnerships to develop, implement, and evaluate the intervention. We expect that this approach will enhance the feasibility of the trial, as well as future dissemination and sustainability of the intervention.

**Trial registration:**

Clinical Trials, NCT02707432. Registered 13 March 2016.

## Background

Cardiovascular disease (CVD) is the leading cause of death in the United States (US) with an estimated 50% of individuals in the US expected to have CVD by 2030 [1–4]. African American residents in rural areas of the south and southeast (also called the “stroke belt”) have the highest prevalence of CVD compared to other populations [5]. Furthermore, CVD prevalence rates for residents of rural areas (13.1%) are higher compared to those in urban areas (11.2%) of the US [6].

In the setting of geographic disparities, striking racial disparities in CVD risk factors, morbidity and mortality are exacerbated. African Americans are disproportionately affected by a higher burden of CVD risk factors: high blood pressure, diabetes, smoking, high cholesterol, and physical inactivity [7, 8]. This heightened risk is prevalent in both youth and older age groups and has increased over time [8, 9]. While CVD disparities at the intersection of race and geography are driven by a disproportionate burden of individual risk factors, racial disparities in rural settings are amplified by associated factors, such as limited access to quality healthcare services, socio-economic burden, dwindling resources, insufficient health infrastructure, and physical barriers to access to care [10, 11].

There are numerous evidence based interventions (EBIs) that have focused on reduction in CVD risk factors. These interventions have often focused on primary prevention of individual risk factors such as hypertension, diabetes, lipids, physical activity, and diet quality [1–4, 12–14]. Many of these have been developed in urban centers, and only a few have included participants that are reflective of geographic and racial disparities in CVD risk. Given the burden of co-morbidities in rural African American populations, interventions targeting only one behavior or risk factor are likely to be less successful than those using intervention components and concepts impacting multiple behaviors [15].

There is a need for culturally adapted interventions that address multiple behavioral risk factors for CVD. Using a Community-Based Participatory Research (CBPR) approach, our team adapted and will implement an EBI to improve cardiovascular risk in African Americans living in a rural underserved setting. This manuscript outlines the design and methods of our study.

## Methods/design

### Description of CBPR partnership

Our research team is comprised of community and academic investigators who are part of Project GRACE (Growing Reaching Advocating for Change and Empowerment) - a community academic research partnership [16]. Project GRACE is guided by CBPR principles and has over a 10-year history of designing and testing interventions. Project GRACE demonstrates both individual and collaborative expertise in community-based CVD outreach, service and research [16]. In prior manuscripts, we have described Project GRACE’s staged approach to partnership development that includes: 1) initial mobilization – inclusion of a larger group of Consortium members who represent a range of institutions and constituencies; 2) establishing organizational structure – development of bylaws and committee structure; 3) capacity building for action – raising individual and group skill level and strengthening partnership capacity; and 4) planning for action – identifying community needs, resources, goals, and planning for intervention implementation [16].

The Project GRACE Steering Committee is responsible for planning and oversight of the research undertaken by the partnership. The Steering Committee is comprised of 26 members with attention to equity across the 2 counties. Six subcommittees conduct project activities: Communications and Publications, Research and Design, Nominating, Bylaws, Events Planning, and Finance. Each subcommittee is chaired by a community stakeholder. This organizational structure ensures a high level of community participation and decision making at every level that builds sustainability, trust, and transparency. The Steering Committee members provide oversight and input into all aspects of the study design, recruitment, implementation and interpretation of findings of studies undertaken by Project GRACE.

For this study, researchers from UNC School of Medicine Center for Health Equity Research partnered with two community-based organizations: Project Momentum, Inc. (PMI) and James McFarlin Community Development, Inc. (JMCD). PMI provides social, physical, and emotional resources for people affected by HIV/AIDS, mental health issues, and substance abuse within Edgecombe, Nash, and Wilson counties of North Carolina. JMCD provides charity for individuals and families as well as resources for community development in Edgecombe and Nash counties.

### Guiding principle for selecting an EBI

Firstly, in planning for this study, we agreed on several factors of importance in selecting an EBI to adapt. First, the EBI should address multiple CVD risk factors and improve CVD risk profiles through lifestyle and behavior modification that include physical activity and dietary change. Given that CVD risk factors often co-occur and rural African Americans have the highest burden of comorbid CVD risk factors, we were interested in interventions that would be applicable to a broad swath of our community.

Secondly, we looked for an intervention that acknowledged or could be adapted to acknowledge the role of the family or important peers in behavior change. Finally, we sought EBIs that enrolled a diverse sample or could be culturally tailored to African American participants either in the initial testing or in subsequent revisions of the program materials. After considering several EBIs that met these criteria, we agreed to adapt PREMIER - a CVD prevention EBI held at four clinical centers that found 12–14% reductions in estimated CVD risk (Johns Hopkins University, Baltimore, MD; Pennington Biomedical Research Center in Baton Rouge, LA; Duke University Medical Center in Durham, NC; and a clinical center at Kaiser Permanente Center for Health Research in Portland, OR) [17]. We decided to name our intervention “Heart Matters.” Heart Matters seeks to reduce weight and improve other CVD risk factors among African American adults age 21 and older with at least one CVD risk factor living in two rural communities.

### Adaptation of the EBI

We adapted PREMIER to fit the needs and resources of our target communities. Community partners would be primarily responsible for implementation. To inform the adaptations made, we used intervention mapping and we categorized the adaptations made using the Wiltsey Stirman framework [18].

### Setting

We will implement Heart Matters in Edgecombe and Nash counties; two counties in eastern North Carolina that are considered rural by urbanized area definitions with respective populations of 54,150 and 93,919 [19]. The two counties share one urbanized area, the city of Rocky Mount. Both have had a large prevalence of African-Americans (56.7 and 40% of population, respectively) that experience significant health disparities with respect to cardiovascular disease [19, 20].

The CVD mortality rates for Edgecombe and Nash counties are 1.23 and 1.13 times the overall rate of North Carolina [5]. Each county experiences high and disparate rates of poverty and CVD [5, 19, 20]. Edgecombe County also has the second highest unemployment rate in the state at 6.9% while Nash County ranked 15th at 5.5% [21]. In Edgecombe County 32.3% of African American residents live in poverty [20]. Similarly in Nash County, 28% of African Americans are live in poverty [19]. Edgecombe and Nash counties report similar trends in CVD prevalence, where CVD and stroke are among the leading causes of death [5]. In community health assessments conducted in the last 3 years, CVD risk factors, such as obesity and hypertension, were among the top 10 health priorities in both counties [19, 22].

### Study design

Heart Matters will be tested using the principles of a randomized stepped wedge design. This pragmatic study design is increasingly used for evaluating the implementation of interventions, particularly for interventions that have already been shown to be effective in more controlled research settings, such as PREMIER. With a high risk population and an available, likely effective intervention, randomized stepped wedge design allows for all participants to receive the treatment, which is an ethical strong point of the design.

We will employ a stepped-wedge design for several reasons: (1) intervention arms can serve as their own control thereby allowing for fewer intervention groups. This is particularly important for implementing EBI in rural settings where resources are limited and time commitment of participants is restricted. (2) The approach is financially pragmatic as it allows for multiple intervention arms to be conducted simultaneously without expending too many resources. (3) This design is uniquely useful in evaluating intervention effects between groups that are receiving the intervention staggered over time and who can serve as controls. (4). The design involves random and sequential crossover of clusters from control to intervention until all clusters are exposed [23].

In the stepped wedge design, all groups experience data collection at baseline. At regular intervals one cluster (or a group of clusters) is randomized to start the intervention and move from “control” to active intervention. By the end of the study, all groups will be exposed to the intervention. Data collection occurs at baseline and at each interval when clusters are exposed to the intervention. In that way, each group contributes observations under both control and active intervention observation periods. Potential challenges of the study design are logistical difficulties with recruitment of the entire study sample of participants during a single time period, simultaneous baseline data collection from all participants, and retention in the study especially during control periods.

To implement Heart Matters, we will cluster individual participants within organizations that we recruit to participate in the study. We will randomize organizations to 2 start times: baseline and 6 months. Randomization by organization reduces the likelihood of data contamination given the fact that rural communities tend to be smaller and participants may be familiar with each other. Though our study only has two start times, a two step design is fairly common [24]. The stepped wedge design will allow for robust data collection in that each group will still contribute observation data at control and intervention time points.

### Heart Matters intervention

Heart Matters uses lifestyle modification to reduce CVD risk factors and includes 26 interactive group sessions and 7 individual visit sessions over 12 months (see Table 1). Heart Matters lifestyle goals include: 1) reducing weight by 15 lbs. or another agreed upon goal, 2) limiting fat intake by consuming 20–50% or less of total calories from fat, 3) limiting daily sodium intake to 2300 mg or less, 4) accumulating 150 min of moderate-intensity exercise each week, 5) limiting alcohol intake; women are advised to consume no more than one alcoholic drink per day and men are advised to consume no more than two alcoholic drinks per day, and 6) diet and physical activity tracking.

**Table 1:** Heart Matters Intervention

Phase	Component	Format	Content
Phase 1: Learning New Information (Months 1–3)	Group Sessions 1–8	• 90 min • Weekly • In-person	• Discuss benefits/barriers to healthy eating and engaging in physical activity • Introduce new or alternative dietary and physical activity behaviors (behavior modifications) • Participate in physical activities and food-tastings
	Individual Sessions 1–3	• 30–60 min • Bimonthly • Via phone	• Set personal goals according to Lifestyle Guidelines • Monitor and track goals • Review food and fitness diaries
Phase 2: Learning New Information (Months 4–6)	Group Sessions 9–14	• 90 min • Bimonthly • In-person	• Discuss benefits/barriers to healthy eating and engaging in physical activity • Introduce new or alternative dietary and physical activity behaviors (behavior modifications) • Participate in physical activities and food-tastings
	Individual Session 4	• 30–60 min • Via phone	• Monitor and track goals • Review food and fitness diaries
Phase 3: Maintenance (Months 7–12)	Group Sessions 15–26	• 90 min • Bimonthly • In-person	• Attend exercise classes • Listen and discuss with guest speakers • Visit restaurants
	Individual Session 5–7	• 30–60 min • Every other month • Via phone	• Monitor and track goals • Review food and fitness diaries

#### Group sessions

Trained facilitators and co-facilitators will conduct group sessions at each organizational site with 15–20 participants per group. Facilitators are members of the community who are teachers, coaches, and clergy members and have 30 min of group supervision of adherence to protocol for each session delivered. Co-facilitators are allied health professionals, such as nutritionists, nurses, and personal trainers. All facilitators participate in an extensive three-part training on research protocols and curriculum.

#### Individual visit sessions

Participants will have seven 60-min individual sessions throughout the intervention. Individual sessions will be pre-scheduled and conducted via phone by the facilitator. Using motivational interviewing techniques, facilitators will discuss participant’s progress and challenges to making behavior changes. Participants develop an individualized action plan at the first individual visit and this plan is revisited in subsequent individual visit sessions in an effort to track individual progress.

## Study procedures

**Fig. 1:**
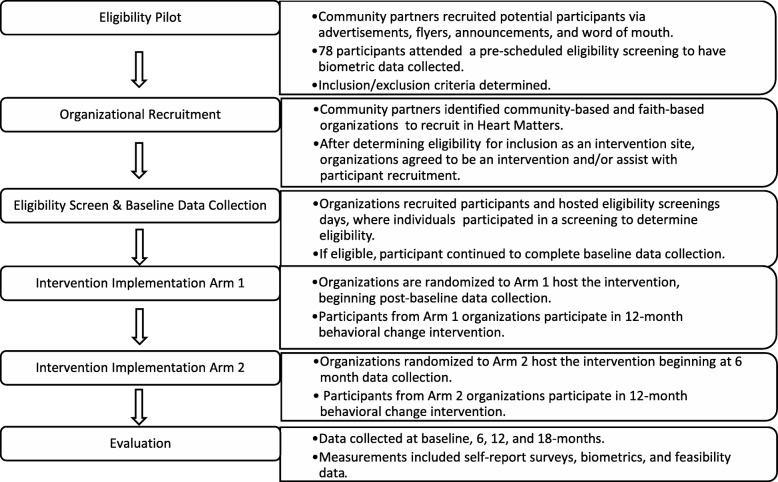
Illustrates the study procedures across time and their major components

Study procedures describe the initial eligibility pilot through the evaluation of Heart Matters (see Fig. 1).

### Pilot to determine eligibility criteria

Local epidemiologic data and the expertise of our community partners suggested the burden of CVD risk was much higher in the Heart Matters communities. We conducted an eligibility screening pilot (*n* = 78) to assess the proportion of individuals that would be eligible based on the criteria used in the PREMIER trial and to determine the inclusion/exclusion criteria for our target population. Eligibility criteria for PREMIER included a systolic blood pressure (BP) of 120–159 mmHg and diastolic BP of 80–95 mmHg, (pre-hypertension or stage 1 hypertension), a body mass index between 18.5–45 kg /m^2^, and excluded individuals with diabetes. For the pilot, participants were recruited by JMCD and PMI community partners via newspaper and radio advertisements, word of mouth, posted flyers, and announcements at faith-based organizations. 59 of 79 screened participants were deemed ineligible using the PREMIER trial criteria.

Based on results of the pilot, we decided to adjust eligibility criteria to include individuals who had been diagnosed by their doctor with diabetes and/or hypertension to ensure Heart Matters was relevant in this community. As such, eligibility was contingent on BMI in range of 18.5–45 kg /m^2^. Full inclusion criteria were: African American, age 21 or older, reside in Nash or Edgecombe counties, self-report at least one CVD risk factor: pre-diabetes or diabetes, hypertension, obesity, family history of early CVD, prior diagnosis of CVD. Exclusion criteria: (a) evidence of active or unstable CVD, or (b) cognitive impairment that limits informed consent.

### Organizational recruitment

We recruited organizations to serve as recruitment and intervention sites from both counties (Edgecombe and Nash). We invited potential organizations based on: (a) existing relationships through the Project GRACE consortium, (b) and a list of organizations beyond existing networks generated by our JMCD and PMI collaborators. Knowing that CVD risk is high in older adult populations, the extended list included senior health and civic organizations. Historically, faith based organizations reach a large segment of the minority community and are a trusted leader in the community. As a result, many organizations were faith-based.

In addition to partnering for participant recruitment, we invited organizations to be intervention sites. We used the following inclusion criteria to evaluate each organization as an intervention site: kitchen space with refrigerator, stove and/or microwave, contact person from each organization available to open and close the building, and layout of how the used space should look at the conclusion of use. We based final inclusion criteria on if the site could host the intervention for all 12 months. Of the original list of criteria, we identified 10 as possible study sites; 8 agreed to participate as intervention sites, and 8 agreed to be recruitment sites. From the 8 recruitment sites, we will recruit individual patrons/members of those organizations to participate in the Heart Matters study.

### Participant recruitment and eligibility screening

Based on sample size calculations (see below), our intended study sample will be 120 participants across both counties. Community-based recruiters at JMCD and PMI will work with organizational leaders to hold recruitment events at each organization. Outreach to individuals will be based on recommendations from organizational leaders and will include direct outreach and information sessions at each organization, distributing written materials/flyers, recruiting at organizational events and exploring the social networks of initial recruits.

### Engagement during control period

We will maintain contact with Arm 2 participants who are not receiving treatment during the initial 6 months. We will use various modes of communication during the delayed treatment phase to boost study retention. We will engage with the participants via text messaging, mailings to their homes, and providing promotional material. Text messages will be sent monthly to each participant. We will send text messages to Arm 2 participants during their initial control period that provide “countdown” related messages, which detail the amount of time until the intervention program begins and express enthusiasm for their continued involvement with the program. Participants will also receive a greeting card via postal mail, either a birthday card or “thinking of you” card for participants who do not have a birthday during the control group period. We will send newsletters to all participants via postal mail twice pretreatment. The length of the newsletter will be two pages and contain content related to African American history, a timely theme (e.g., spring cleaning), and associated give-away prize. We will randomly select one prize winner from each organization represented in the Arm 2 group. Finally, each participant will receive a magnet with the project logo in an effort to maintain the visibility of the project during the control period. We will strategically time these modes of contact across the months of delayed treatment.

### Informed consent process

We will obtain verbal informed consent from potentially eligible participants for the initial screening during which individuals will provide identifiable information (e.g. contact information). After the eligibility screening, we will obtain written informed consent for each eligible participant who decides to enroll in the study. Community partners at JMCD and PMI will be responsible for enrolling participants into the study. This study was reviewed and approved by the UNC Institutional Review Board and any subsequent modifications will undergo same process. The data monitoring, management and safety operations are overseen by the Scientific Advisory Board, a recommending body of external, expert investigators that conducts annual reviews, including data on adverse events. Any recommended changes will be communicated to PI to modify protocols with IRB approval, and then re-consenting participants when needed.

### Randomization process

FL, the statistician, will randomize organizations into Arm 1 (immediate intervention delivery) and Arm 2 (6 month delayed intervention delivery) under a matched pair in order to balance the sample size between Arm 1 and 2. Organizations will be paired based on the number of participants, and FL will randomly assign one organization from each pair to each arm. We do not anticipate such a process of pairing will confound any clustering effect unlike a conventional matched pair design.

### Incentives for individuals and organizations

Participants will receive up to $195 total in incentives for participation in study procedures. Participants will receive a tiered compensation: $5 for completing the eligibility screening, $25 for baseline data collection, $15 for 6 month data collection, $20 for 12 month data collection, and $20 for 6 month post intervention data collection. Throughout the intervention, participants will receive a $15 cash incentive for regularly attending the sessions. We will be providing incentives at Group Sessions 4, 8, 12, 16, 20, 24, and 26. Additionally, participants will be given Heart Matters branded items throughout the intervention (e.g. water bottles).

We will use a tiered structure for organizational compensation based on level of participation in the study. Organizations that agree to help with recruitment of individual participants will receive $50 for each enrolled participant. Organizations that agree to serve as an intervention site will receive $1000. Organizations that both agree to help with recruitment of participants and serve as a host site for intervention implementation will receive $2000.

### Data collection procedures

Immediately following the consent process, eligible individuals will complete the baseline data collection in a community setting. All data collection is completed by trained research staff from the partner community organizations, JMCD and PMI. Data collection will include biometric and biomarker data as well as self-report behavioral data. Trained research staff will collect biometrics such as weight, height, grip strength, and balance. Trained research staff who are registered nurses will collect blood pressure, hemoglobin A1c, and blood spot data. Study staff will record the biometric data on paper forms which will be entered into REDCap, a secure web-based application for data management. For participants that opt to consent to biomarker data collection in separate consent process, we will transport biomarker data to the UNC Biospecimen processing facility for storage and analysis. For participants with life-threatening A1c levels, they will be immediately transported to emergency services, and adverse event data will be documented by JMCD and PMI research staff.

Trained data collectors will conduct the structured interviews. The baseline questionnaire will include behavioral measures and structured questions on demographics (e.g. age, race, marital status, SES), health history, medications, CVD risk factors and behaviors, knowledge, quality of life, diet, exercise, self-efficacy, and social support (see Table 2). Data collectors will enter data from the paper questionnaire forms into REDCap.

**Table 2:** Heart Matters Primary & Secondary Outcomes

Outcome	Measure	Data Collection Method	Data Collection Time Points		
			Baseline	6 mo.	12 mo.
Primary	Body Weight (lbs.)	Tanita WB-800 Professional Digital Weight Scale	x	x	x
Secondary	Blood Pressure	Omron HEM907XL-Automatic Digital Blood Pressure Monitor	x	x	x
	Grip Strength	Camry 200 Handgrip Dynamometer	x	x	x
	Balance	Timed Up and Go Test	x	x	x
	Blood Glucose (A1c)	A1CNow Plus System	x	x	x
	Cardiovascular Inflammatory Biomarkers	Whatman™ 903 Protein Saver Blood Spot Collection Card	x	x	x
	Diet	FLASHE Dietary Screener	x	x	x
	Physical Activity	7-day Physical Activity Recall Questionnaire	x	x	x
	Self-Efficacy	Self-Efficacy for Diet and Exercise Behaviors Questionnaire	x	x	x
	Social Support	Social Support for Diet and Exercise Behaviors Questionnaire	x	x	x
	Health and Well-Being	SF-36 Questionnaire	x	x	x
	CVD Risk Factors	Life’s Simple 7 Questionnaire	x	x	x

Throughout the study, data collectors and intervention facilitators will also collect process (recruitment, reach, and fidelity) and feasibility (demand, acceptability, and practicality) data about Heart Matters. Data collectors will enter quantitative data (logs, checklists, and questionnaires) into REDCap, and we will send qualitative focus group and interview audio data to a transcription service (see Table 3).

**Table 3:** Heart Matters Implementation Process & Feasibility Outcomes

Outcome	Measure	Data Collection Method	Data Collection Time Points			
			Base-line	6 mo.	12 mo.	Each Session
Process	Recruitment	Data Logs	x			x
	Reach	Data Logs	x			x
	Fidelity	Checklist				x
Feasibility	Demand	Focus Groups				x
	Acceptability	Survey & Focus Groups			x	
	Practicality	Focus Groups & Key Informant Interviews			x	

## Heart Matters outcomes and measures

### Primary outcome

#### Body weight

At each data collection point, data collectors will collect the primary outcome, weight. For a complete list of measures, please refer to Tables 2 and 3.

### Secondary outcomes

#### Blood pressure

At each data collection point, registered nurses will take three blood pressure readings while the participant is in a seated position with their arm outstretched. We will use the average of three readings as the measurement for the specified data collection period. We will use the JNC-8 Hypertension Guideline Algorithm as our guide for defining controlled and uncontrolled BP [25].

#### Cardiovascular inflammatory biomarkers

Registered nurses will collect CVD inflammatory biomarkers: Insulin, C reactive protein, Triglycerides, Cholesterol (Total, HDL and LDL), Interleukin-6 (IL-6) and Tumor Necrosis Factor using a standard blood spot data collection protocol [26].

#### Grip strength

Data collectors will obtain three grip strength readings from the participant while they are standing and select the highest measurement out of 3 trials. Comparing participant’s score with analogous age and gender normed data will indicate a participant’s level of strength [27].

#### Balance

Data collectors will use the Center for Disease Control TUG Test Protocol to conduct the balance assessment [28, 29]. A completion time of 12 s or more indicates the individual is at risk of falling. The TUG has been shown to be both reliable and valid [30, 31].

#### Blood glucose

Registered nurses will assess participants’ hemoglobin A1c blood glucose levels using the A1CNow Plus System. Aligned with the American Diabetes Association, a participant with a hemoglobin A1c > 7% will be considered to have uncontrolled diabetes [32].

#### Diet

This nutritional self reported assessment is based on the nutritional and dietary assessment used in the Family Life, Activity, Sun, Health, and Eating (FLASHE) Study that is sponsored by the National Cancer Institute [33]. It is a self-assessment survey about dietary intake in the past 7 days. Items are grouped by similarities in healthfulness and type. Participants will answer questions about their beverage and food intake.

#### Cardiovascular disease risk factors

My Life Check -Life’s Simple 7 tools are self-assessment measures developed by the American Heart Association (AHA) based on the AHA 2020 impact goals. The questionnaire is based on the definition of ideal cardiovascular health that includes non-smoking, BMI < 25, physical activity at goal levels, diet consistent with current guidelines, untreated total cholesterol < 200 mg/dL, untreated blood pressure < 120/80, and fasting blood glucose < 100. Cardiovascular health status using these metrics is then defined as poor, intermediate, or ideal. Ideal CV health is defined as the presence of optimal levels of all seven metrics and no clinical evidence of CVD.

#### Physical activity

To assess level of physical activity, we will use the 7-day Physical Activity Recall Assessment [34]. The self-reported measure assesses the amount and intensity of physical activity an individual participants in over a seven-day period. The survey also asks participants to gauge their physical activity in relation to others of the same age and sex on a scale of 1 (extremely inactive) to 7 (extremely active).

### Implementation process and feasibility outcomes

#### Process

We will use Steckler and Linnan’s process evaluation framework to assess indicators of process (recruitment, reach, and fidelity) throughout the implementation of the intervention using qualitative and quantitative data collection methods [35]. Specifically, we will use recruitment rates, retention rates and reach logs to measure how many participants were *reached* for participation. For example, we will assess how many were *recruited* in each arm of the study; how many dropped out and at which point; and, how many completed the study. We will use an implementation checklist to assess *fidelity* of the adapted EBI implementation throughout the intervention. We will collect data at facilitated group sessions to document to what extent the protocols are followed and whether the core components of the intervention are delivered as planned.

#### Feasibility

We will assess Bowen et al. (2009) indicators of feasibility (demand, acceptability, and practicality) throughout the implementation of the intervention and at 12 months for both arms of the intervention using quantitative and qualitative measures [36]. We will assess *demand* by comparing frequency and patterns of participants’ use of recommended health strategies outside of sessions, which were taught in the previous session. We will collect *acceptability* data from the participants to determine the intervention satisfaction. We will assess *practicality* by conducting key informant interviews with the intervention facilitators to determine environmental influences, facilitators, and barriers they experienced while implementing Heart Matters and what modifications they made to adapt the intervention. Similarly, we will assess *practicality* using qualitative focus groups with a subsample of Heart Matters participants to determine environmental influences, facilitators, and barriers related to their participation in Heart Matters.

### Sample size

Our sample size calculation is based on the effect size in weight change, our primary outcome, reported by the PREMIER trial [19]. With 1.8 kg difference in weight changes (SD = 3.2) between intervention and control groups, we need 102 participants in total to reach 80% power with 2-sided **α** = 0.05. However, considering the stepped wedge design as a special case of clustered randomized trials, the sample size was further inflated to 140 when the intra-class correlation coefficient (ICC) among members in the same organization was equal to 0.01. Further 15% of initial recruits may drop out, so our targeted number of recruits is set at 120. Aligned with PREMIER results, we hypothesized there will be a significant difference in weight change between control data observations and active treatment data observations from baseline to 12-months. We calculated the power using stepped wedge command in STATA/SE 12.1 (College Station, TX). The detail are described in Hemming and Girling [37].

### Analysis plan

To acknowledge the stepped wedge design is not a completely randomized trial for individual subjects, we will first compare the characteristics at the individual level, as well as in the organization level to account for possible unbalance of initial assignments. Means, standard deviations, frequencies and proportions will be compared between assignments using appropriate statistical tests, mainly t-tests for continuous variables and chi-squared tests for categorical variables. If the continuous variable is not normally distributed, we will use a non-parametric test such as Mann-Whitney to test the difference. We will use a linear mixed effects model to analyze the primary outcome recorded at baseline and every 6 months to account for the natural dependence between individuals from the same organization and repeated measurements from the same subject. The model will include an indicator to detail whether the outcome was collected during the intervention or control period, and include those characteristics that were not balanced in the first step for adjustment. We will also test whether the outcome after the intervention period of the first step group was different from the one in other periods to access the carryover effect of the intervention. All of the analysis will be implemented using SAS 9.3 (Cary, NC). A *p*-value smaller than 0.05 will be considered statistically significant.

## Discussion

The implementation of Heart Matters is innovative in that it takes a social ecologic approach to advancing individual CVD risk reduction in rural minority communities. We aim to shift the paradigm of how CVD risk prevention EBIs are tested and adapted using a CBPR approach that engages community members as full partners in the design and implementation of the trial. End users of the products of the research are engaged at all levels, thus enhancing dissemination and sustainability. The expected outcome of this research is a model of successful translation of an effective EBI in a rural minority community and identification of how best to support its implementation. In addition to advancing the science of CVD prevention research, we also expect to have a positive impact on preventing CVD morbidity in rural minority communities. Our approach will advance the literature on feasibility of adapted EBIs in rural communities that are at an increased risk for CVD. Furthermore our study will identify social and physical environmental factors that influence effective adaptation and implementation of CVD EBIs such as PREMIER.

## Abbreviations

CBPR: Community-based participatory research
CVD: Cardiovascular disease
EBI: Evidence-based intervention
